# Dietary Phytogenics and Galactomannan Oligosaccharides in Low Fish Meal and Fish Oil-Based Diets for European Sea Bass (*Dicentrarchus labrax*) Juveniles: Effects on Gill Structure and Health and Implications on Oxidative Stress Status

**DOI:** 10.3389/fimmu.2021.663106

**Published:** 2021-05-12

**Authors:** Silvia Torrecillas, Genciana Terova, Alex Makol, Antonio Serradell, Victoria Valdenegro-Vega, Marisol Izquierdo, Felix Acosta, Daniel Montero

**Affiliations:** ^1^ Grupo de Investigación en Acuicultura (GIA), IU-ECOAQUA, Universidad de Las Palmas de Gran Canaria, Las Palmas, Spain; ^2^ Department of Biotechnology and Life Sciences, University of Insubria, Varese, Italy; ^3^ Delacon Biotechnik GmbH, Global Solution Aquaculture Unit, Engerwitzdorf, Austria; ^4^ Biomar A/S, Global RD Health. BioMar AS, Trondheim, Norway

**Keywords:** European sea bass, gill morphology, oxidative stress, functional diets, prebiotics, phytogenics, low FM/FO diets

## Abstract

An effective replacement for fish meal (FM) and fish oil (FO) based on plant-based raw materials in the feed of marine fish species is necessary for the sustainability of the aquaculture sector. However, the use of plant-based raw materials to replace FM and FO has been associated with several negative health effects, some of which are related to oxidative stress processes that can induce functional and morphological alterations in mucosal tissues. This study aimed to evaluate the effects of dietary oligosaccharides of plant origin (5,000 ppm; galactomannan oligosaccharides, GMOS) and a phytogenic feed additive (200 ppm; garlic oil and labiatae plant extract mixture, PHYTO) on the oxidative stress status and mucosal health of the gills of juvenile European sea bass (*Dicentrarchus labrax*). The experimental diets, low FM and FO diets (10%FM/6%FO) were supplemented with GMOS from plant origin and PHYTO for 63 days. GMOS and PHYTO did not significantly affect feed utilization, fish growth, and survival. GMOS and PHYTO downregulated the expression of *β-act*, *sod*, *gpx*, *cat*, and *gr* in the gills of the fish compared with that in fish fed the control diet. The expression of *hsp70* and *ocln* was upregulated and downregulated, respectively, in the GMOS group compared with that in the control group, whereas the expression of *zo-1 was* downregulated in the PHYTO group compared with that in the GMOS group. The morphological, histopathological, immunohistochemical, and biochemical parameters of the fish gills were mostly unaffected by GMOS and PHYTO. However, the PHYTO group had lower incidence of lamellar fusion than did the control group after 63 days. Although the tissular distribution of goblet cells was unaffected by GMOS and PHYTO, goblet cell size showed a decreasing trend (−11%) in the GMOS group. GMOS and PHYTO significantly reduced the concentration of PCNA+ in the epithelium of the gills. The above findings indicated that GMOS and PHYTO in low FM/FO-based diets protected the gill epithelia of *D*. *labrax* from oxidative stress by modulating the expression of oxidative enzyme-related genes and reducing the density of PCNA+ cells in the gills of the fish.

## Introduction

The interaction between gill mucosal epithelia and the external environment is complex and highlights the importance of this tissue as an organ of defense against a wide range of hazards, including stress and physicochemical and biological alterations (1, 2). Gill epithelia facilitate respiration by separating blood circulation from external media through an exceptionally thin epithelium, which is composed mainly of two cell types, pavement cells (PVCs) and mitochondria-rich cells (MRCs) (3). PVCs provide physical protection and are involved in gas and osmotic exchange, as well as in acid–base regulation (1, 4–6). MRCs are involved in active ion transport and regulation, gas exchange, and maintenance of blood acid–base balance (1). These cells are characterized by a mitochondria-rich cytoplasm and the presence of an extensive tubulovesicular system located in the apical region of the cell, where the Na^+^/K^+^-ATPase transporter enzyme is located (6, 7). When challenged, both types of cells can gain functionality with respect to their structural roles and anatomical localizations (3). Gill associated lymphoid tissue (GIALT) is composed mainly of B cells, T cells, eosinophilic granulocytes, macrophages, neutrophils, and goblet cells, and contains several humoral immune-related molecules (2, 8). Like other mucosal immune systems, the gill has an associated microbiota; however, because of the nature of this tissue, microbial colonization seems to be restricted to protected areas (gaps between pharyngeal arches and lamellae) (9).

Reared fish are exposed to several natural and induced stressors, some of which produce reactive oxygen species and nitrogen species (RONS), which can induce physiological and morphological alterations (10), especially in susceptible mucosal tissues, including fish gills. Histopathological gill lesions are considered a good indicator of oxidative stress-inducing factors such as xenobiotics (11–15).

Oxidative stress results from an imbalance between the levels of RONS and the capacity of the antioxidant defense system to cope with them (16). This imbalance triggers the oxidation of essential biomolecules and favors multiple disease conditions (17). Similar to higher vertebrates, the methods for fish antioxidant defenses include enzymatic mechanisms and non-enzymatic endogenous and exogenous compounds (18). Superoxide dismutase (SOD), catalase (CAT), and glutathione peroxidase (GPX) are the main antioxidant enzymes that constitute the first line of defense against oxidative stress and have been traditionally used as indicators of the antioxidant status of an organism (19). Moreover, proteins, such as heat shock proteins (HSPs), play a cytoprotective role against oxidative stress by initiating protein folding, repair, and degradation of irreparable proteins, as well as regulating the endogenous generation of reactive species *via* a modulation of the inflammatory cascade and intrinsic apoptosis processes (20, 21). However, an increase or inhibition of antioxidant activity can vary depending on the intensity and duration of the stressors, as well as on the susceptibility of the exposed fish species (19, 22).

Non-enzymatic endogenous antioxidants, such as uric acid, tripeptide glutathione, or melatonin (23), and exogenous antioxidants can be ingested through the diet or as dietary supplements. Additionally, some dietary compounds that do not directly neutralize reactive species, but rather enhance endogenous enzymatic activity, may also be classified as antioxidants (24).

Under some oxidative/nitrosative stress conditions, such as those attributed to intensive fish culture practices, dietary supplementation with exogenous antioxidants such as vitamins, flavonoids, carotenoids, plant polyphenols, allyl sulfides, curcumin, melatonin, or polyamines may offer a good functional strategy to maintain proper levels of RONS (23, 25, 26). Although most of the studies investigating fish oxidative stress are related to the effects of xenobiotics or pollutants, the use of functional additives, which are capable of triggering the fish antioxidant system, has gained attention during the last decade in aquaculture because of its potential benefit in preventing diseases and promoting health (17, 27–32).

Among these additives are phytogenic feed additives (PFA), consisting of highly active plant substances that encompass much more than essential oils because they include phytochemical compounds such as saponins, flavonoids, mucilages or tannins (33). A wide variety of products and extracts are available; garlic and labiate plant essential oils have been proven to have antioxidant properties (34) in higher vertebrates (35–38) and fish (39–43). These antioxidant capacities are not limited to functional PFA compounds, as several authors have observed antioxidant properties in other functional additives such as prebiotics, particularly glucomannans (19, 44).

However, little is known about how these dietary functional additives may affect the oxidative status of fish gills and their associated GIALT function, particularly when they are included in plant-based fish diets. Thus, the aim of the present study was to determine the effects of galactomannan oligosaccharides of plant origin (GMOS) and a PFA (mixture of garlic oil and labiatae plant extracts) on the oxidative stress status and mucosal health of the gills of juvenile European sea bass when supplemented in low FM/FO diets. For this purpose, structural, cellular, and oxidative dietary-associated changes were evaluated using gene expression analyses and optical and electron microscopy studies.

## Materials and Methods

### Diets

Three low fish meal (10%FM) and fish oil (6%FO)-based diets were formulated according to commercial standards. One of the experimental diets was void of the test ingredients (control diet), while the remaining two contained 5,000 ppm of galactomannan oligosaccharides (GMOS; Delacon, Austria) and 200 ppm of a blend of garlic and labiatae-plant oils (PHYTO; Delacon, Austria), respectively. The diets were formulated to meet the nutritional requirements of European sea bass. The specific composition of PHYTO and GMOS cannot be disclosed due to confidentiality issues because these functional additives are a prototype of potential commercial products (Delacon internal product code: SBPMH01 and SBPMH02, respectively).

The isoenergetic and isonitrogenous diets were industrially produced in the BioMar Tech-Centre (Brande, Denmark) using an extrusion process. To ensure product stability, GMOS was included in the mix during the pre-extrusion process, whereas PHYTO was homogenized with dietary oil and included by vacuum coating during the post-extrusion process. The stability of the functional products was checked before the production of the diet and at the beginning of the feeding trial. The ingredients used in the diets and their proximate composition are detailed in **Table 1**.

**Table 1:** Main ingredients and analyzed proximate composition of the diets.

Ingredients (%)	DIETS		
	CONTROL	GMOS	PHYTO
Fish meal^1^	9.6	9.6	9.6
Soya protein concentrate	18.2	18.2	18.2
Soya Meal	11.6	11.6	11.6
Corn gluten meal	24.1	24.1	24.1
Wheat	8.5	8,0	8.5
Wheat gluten	1.9	1.9	1.9
Guar Meal	7.7	7.7	7.7
Rapeseed extracted	3.0	3.0	3.0
Fish oil^2^	6.5	6.5	6.5
Rapeseed oil^3^	5.2	5.2	5.2
Vitamin and mineral premix^4^	3.6	3.6	3.6
Antioxidant^5^	0.06	0.06	0.06
Galactomannan oligosaccharides^6^	0	0.5	0
Phytogenic^7^	0	0	0.02
**Proximate composition (% of dry matter)**			
Crude lipids	19.91	20.44	20.47
Crude protein	49.30	49.27	49.76
Moisture	5.10	5.01	5.06
Ash	7.02	6.41	6.49
Gross Energy (MJ/kg, as is)^8^	22.07	22.11	22.17

^1^South-American, Superprime 68% (63-68% protein; 8-9.5% lipids). ^2^South American fish oil. ^3^DLG AS, Denmark. ^4^Vilomix, Denmark. ^5^BAROX BECP, Ethoxyquin. ^6^Delacon Biotechnik GmbH, Austria. ^7^Delacon Biotechnik GmbH, Austria. ^8^Determined using a calorimetric bomb (Eurofins Food & Feed testing, Norway, AS).

### Experimental Conditions

The study was carried out at the facility of the Parque Científico-Tecnológico Marino (PCTM), University of Las Palmas de Gran Canaria (Telde, Canary Island, Spain). Healthy juvenile European sea bass were procured from a local farm (Aquanaria, Castillo del Romeral, Gran Canaria, Canary Islands, Spain). The fish were acclimatized for four weeks to the new environment (6.6–6.1 ppm dissolved O_2_, 18.2–20.2°C). Thereafter, the fish (mean weight: 23.02 ± 0.67 g; mean length: 11.89 ± 0.15 cm) were randomly assigned to nine 500 L (75 fish/tank) fiberglass tanks at an initial density of 3.5 kg·m^3^. The tanks were equipped with an open flow-through water system and the fish were exposed to natural photoperiod (12L:12D). The fish were fed three times a day to apparent satiation six days a week for 63 days. Before final sampling, each fish was anesthetized in accordance with the regulations of the European Union Directive (2010/63/EU) and Spanish legislation (RD 53/2013) for animal experiments. Fish handling was performed under natural clove oil anesthesia (0.2 ml/L; Guinama S.L; Spain, Ref. Mg83168), and discomfort, stress, and pain to the experimental animals were avoided as much as possible during the experiment. For sampling, fish were euthanized using an overdose of natural clove oil (5 ml/L; Guinama S.L; Spain, Ref. Mg83168).

The second and third holobranch from the right basket of the gill (n = 4 fish/tank) were stored in 4% paraformaldehyde at 4 °C for 48–72 h before being embedded in paraffin for morphological and immunohistochemical studies. Portions of the same holobranchs (central region) were fixed at 4°C in 2.5% glutaraldehyde and 0.15 M HEPES buffer (pH = 7.4) for structural studies (n = 3 fish/tank). Sections of the second and third holobranch from the right basket of the gill were sampled (n = 4 fish/tank) and stored at −80°C for gene expression analysis. The Bioethical Committee of the University of Las Palmas de Gran Canaria approved all the protocols used in the present study (approval no. 007/2012 CEBA ULPGC).

### Proximate Compositions and Gross Energy of Diets

The proximate compositions of the feed were analyzed according to standard procedures (45). Dry matter content was determined after drying in an oven (110°C) to constant weight, and ash content was determined by combustion in a muffle furnace (600°C, 12 h). The crude lipids were extracted as described in Ref. (46) and crude protein content was determined (N × 6.25) using the Kjeldahl method. The gross energy content of diets was determined using a calorimetric bomb (Eurofins Food & Feed testing, Norway, AS).

### RT-qPCR Analysis

Total RNA was extracted from the gill samples using TRI-Reagent (Sigma-Aldrich, Sant Louis, MO, USA) and RNeasy^®^ mini-Kit (QIAGEN, Germany) according to the manufacturer’s instructions. The RNA was quantified by spectrophotometry (Nanodrop 1000, Thermo Fisher Scientific Inc., USA) and its integrity evaluated on a 1.4% agarose gel with Gel Red™ (Biotium Inc., Hayward, CA, USA).

Total RNA was reverse transcribed in a 20 μl reaction volume containing 2 μg total RNA, using a ThermoScript TM Reverse Transcriptase kit (Invitrogen, California, USA), until cDNA was obtained in a thermocycler (Mastercycle ^®^ nexus GSX1, Eppendorf AG, Hamburg, Germany) run according to the manufacturer’s instructions. The samples were then diluted to 1:10 in MiliQ water and stored at −20°C.

An aliquot of cDNA obtained by reverse transcription was then amplified by Real Time-qPCR using a designed primer set for each gene. The target genes were: *superoxide dismutase* (*sod*), *glutathione peroxidase (gpx), glutathione reductase (gr), catalase (cat), heat shock protein 70 kilodaltons (hsp70), zonula occludens 1 (zo-1)* and *occludin (ocln)*. Primers for the target genes and for the housekeeping gene (*α-tubulin*) were designed based on the cDNA sequences available in the GenBank database for *Dicentrarchus labrax*. The GenBank accession numbers and primer sequences are listed in **Table 2**.

**Table 2:** Primers used for RT-qPCR.

Gene	Accession number	Primer	Nucleotide sequence (5’- 3’)
*sod*	FJ860004.1	Forward	CATGTTGGAGACCTGGGAGA
		Reverse	TGAGCATCTTGTCCGTGATGT
*gpx*	FM013606.1	Forward	AGTTCGTGCAGTTAATCCGGA
		Reverse	GCTTAGCTGTCAGGTCGTAAAAC
*gr*	FM020412.1	Forward	CACTCGTCACCAAAGACCCA
		Reverse	GCAATGGCAACAGGTGTGAG
*cat*	FJ860003.1	Forward	TGGGACTTCTGGAGCCTGAG
		Reverse	GCAAACCTCGATCGCTGAAC
*hsp70*	AY423555.2	Forward	GGACATCAGCCAGAACAAGAGA
		Reverse	GCTGGAGGACAGGGTTCTC
*zo-1*	MH321323.1	Forward	CGGCCTGCAGATGTTCCTAA
		Reverse	GCTGAGGGAATTGGCTTTGA
*ocln*	MH321322.1	Forward	GGACGAAGACGACAACAACGA
		Reverse	CCATGGGAGAAAGCCTCTGA
*α-tub (hk)*	AY326429.1	Forward	AGGCTCATTGGCCAGATTGT
		Reverse	CAACATTCAGGGCTCCATCA

hk, housekeeping.

The Real Time-qPCR was carried out in a final volume of 20 μl, containing 3 μl of cDNA (100 ng), 10 μl of iTaq Universal SYBR^®^ Green Supermix (Bio-Rad, Milan, Italy), and 500 nM of each primer, using CFX96 real-time PCR instrument (Bio-Rad, Milan, Italy), according to the manufacturer’s instructions. The reaction thermal conditions were as follows: 95°C for 1 min, then 40 cycles at 95°C for 10 s, and 60°C for 30 s. A blank sample containing nuclease-free water instead of the cDNA template was included in each assay as a negative control. Relative expression levels were calculated using the 2^−ΔΔCt^ method (47), and *α-tubulin* as housekeeping gene.

For the ΔΔCt calculation to be valid, the amplification efficiencies of the target and reference gene must be approximately equal. For this, The CFX Maestro™ Software (Biorad) was used to calculate the efficiency (E) of each primer set (**Supplementary Figure 1**). The same software allowed the selections of the appropriate reference gene based on the average M value and analyzes gene stability by means of the reference gene selection tool (CFX Maestro™ Software User Guide Version 1.1).

To calculate the expression data, we related the PCR signal of the target transcript in the treatment groups (GMOS and PHYTO) to that of samples of an untreated control (CTRL), which was considered as one.

### Morphological Studies

For morphological examinations, 4 µm transverse and sagittal gill sections were stained with hematoxylin and eosin (H&E) for optical examination, with Alcian Blue (pH = 2.5) for goblet cell differentiation, and with May–Gr̈nwald Giemsa (MGG) to study leukocyte distribution and presence (48). Digital images of the slides were obtained using Olympus VS120 digital scanner (Optic system BX61VS, Tokyo, Japan) equipped with VC50 and VS-XM10 cameras and were processed with Olympus VS software (VS-NIS-SQL-V2.6, Tokyo, Japan).

#### Histopathological Examination of Gill

Histopathological examination of the gills was performed on H&E-/MGG-stained sections (n = 12 fish per diet). The effects of GMOS and PHYTO on the incidence of fish lamellar fusion or branching, lamellar telangiectasis/aneurysms, chloride cell hyperplasia, as well as on the inflammation status and leukocyte-infiltrated populations of the gills were evaluated. The examiners were unaware of the experimental treatments, and assessments were based on a previously established semiquantitative scoring system as follows: 1 (weak), 2 (moderate), and 3 (high).

#### Gill Mucus Production Studies

Digitalized images of gill transverse sections (Alcian Blue pH = 2.5) were used to determine the morphological characteristics of gill goblet cells. For that purpose, CellSens Dimension Desktop 1.16 (Olympus Iberia, Spain) was calibrated to determine the area (μm^2^), minimum diameter, and minimum perimeter (μm) of the goblet cell for each transverse section of the gill. Sagittal sections stained with Alcian Blue (pH = 2.5) were used to determine the location of the goblet cells along the primary lamellae and interlamellar spaces.

### Gill Immunohistochemistry

Gill sagittal and transverse sections were routinely dewaxed and rehydrated. After antigen retrieval (High pH (Dako, Denmark) endogenous peroxidase activity was blocked with peroxidase blocking solution (Dako, Denmark). The sections were incubated in a humid chamber overnight at 4°C with primary rabbit antibody anti-iNOS-2 (diluted 1:200; Santa Cruz Biotechnology, CA, USA), and anti-PCNA (diluted 1:200; Thermo Fisher Scientific, CA, USA). Immunohistochemical staining was performed using a horseradish peroxidase (HRP) anti-rabbit EnVision System (Dako, Denmark) with diaminobenzidine (Dako, Denmark) or with 3-amino-9-ethylcarbazole (AEC+) chromogenic substrate system (Dako, Denmark). Negative controls were processed by replacing primary antibodies with a primary antibody diluent. Immunopositivity for anti-iNOS-2 was evaluated semi-qualitatively according to a previously established immunohistochemical scoring system with immunolabeling intensity grades as follows: 1 (weak), 2 (moderate), and 3 (strong) for each area/cell evaluated. All slides, except for the negative controls, showed immunoreactivity to the antibodies assayed. Immunopositivity evaluation for PCNA-positive cells was carried out using the CellSens Dimension Desktop 1.16 software (Olympus Iberia, Spain), which was calibrated to determine the percentage of gill area covered by positive PCNA cells for each transverse section. None of the negative controls showed any immunoreactivity.

### Gill Ultrastructure Examination

Gill transverse primary lamellar samples were post-fixed at 4°C in 2% osmium tetroxide and 2% uranyl acetate, dehydrated, and embedded individually in an Embed 812 (Electron Microscopy Sciences (EMS), PA, USA) resin block. Semi-thin serial transverse sections (n = 3–5 per individual fish) were contrasted with toluidine blue and examined under a light microscope (49). Ultrathin (50 nm) sections were qualitatively evaluated using a JEOL JEM-1011 transmission electron microscope (TEM; JEOL USA, Inc., USA) equipped with a digital camera MegaView III soft imaging system CCD camera (EMSIS GmbH, Germany). Cellular membrane lining appearance, primary and secondary lamellar structure, cellular morphology, TJ structure, microridge structure, mitochondria-rich cells (chloride cells) microvilli, and the number of infiltrated leukocytes were the main parameters evaluated.

### Statistical Analyses

Statistical analyses were performed following the methods described in Ref. (50). All data were tested for normality and homogeneity of variance. Differences were considered significant at p ≤0.05. Differences between dietary treatments were determined using one-way ANOVA. When F values were significant, individual means were compared using *post hoc* Tukey or Games–Howell tests for multiple means comparison. When required, data arcsine square root transformation was performed, particularly for data expressed in percentage (51). Morphological and immunohistochemical findings, which were based on a range scale evaluation, were analyzed by paired comparisons (Mann–Whitney U test). Analyses were performed using the SPSS Statistical Software System v21.0 (SPSS, Chicago, IL, USA) and GraphPad Prism 8 software (GraphPad Software, Inc., La Jolla, CA, USA).

## Results

### Growth Parameters

At the end of the feeding trial, dietary supplementation with the additives did not affect (p >0.05) fish growth and diet utilization, as described previously in Ref. (52). Mortality was negligible (<1%) and not associated with a specific diet.

### RT-qPCR Analyses

**Figure 1:**
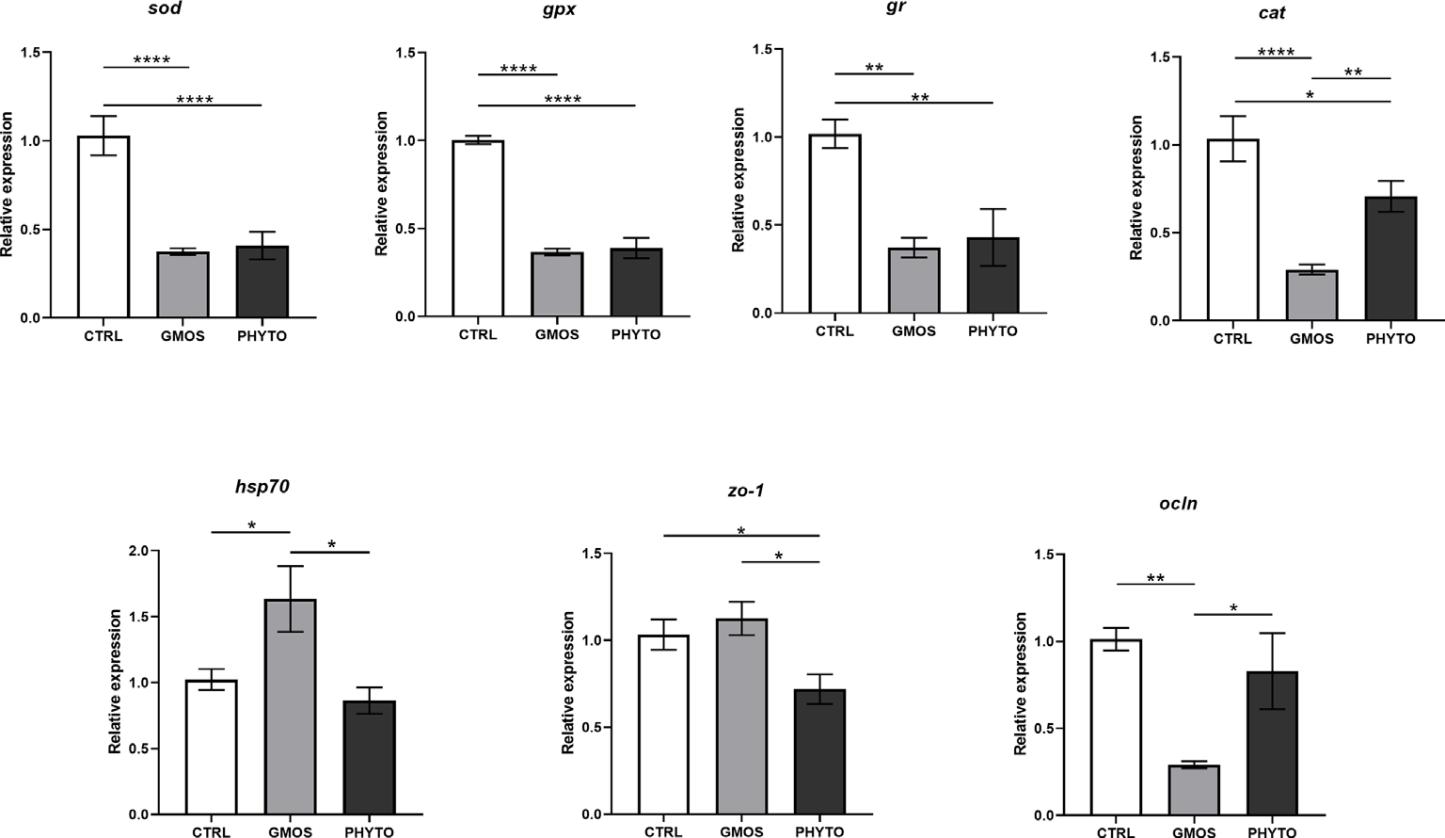
Expression levels of genes of interest in gills of *Dicentrarchus labrax* samples. Diets: CTRL (control diet), GMOS (5,000 ppm galactomannan oligosaccharides), PHYTO (200 ppm phytogenic). (****p-value < 0.001, **p-value < 0.01, *p-value < 0.05; one-way ANOVA; Tukey).

The RT-qPCR showed that the expression of the target genes was significantly different in the different diet groups (**Figure 1**). The expression of *sod*, *gpx*, and *gr* decreased by half in fish fed the GMOS and PHYTO supplemented diets compared with the expression level in fish fed the control diet. Similarly, the expression of *cat* in the gills of fish fed the supplemented diets were significantly lower compared with that in fish fed the control diet, with the GMOS group recording the lowest expression. Contrarily, the expression of *hsp70* was significantly higher in fish fed the GMOS supplemented diet compared with that in fish fed the control and PHYTO supplemented diets. Fish fed PHYTO supplemented diet recorded significantly lower expression level of *zo-1* compared with that in fish fed the control and GMOS diets, whereas the expression of *ocln* was significantly downregulated in the gills of sea bass fed GMOS supplemented diet compared with that in fish fed the control and PHYTO supplemented diets.

### Morphological Studies

#### Histopathological Examination of Gill

**Figure 2:**
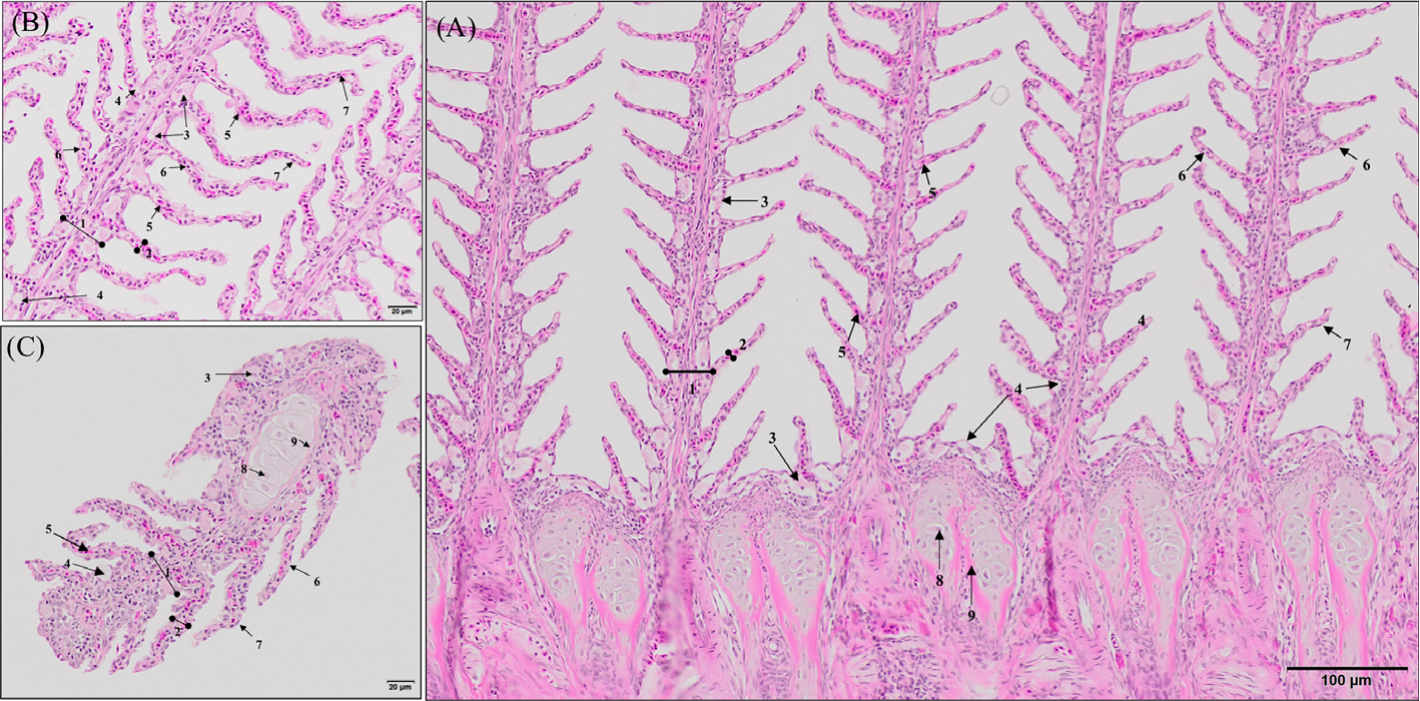
General morphology of European sea bass (*Dicentrarchus labrax*) gill. **(A, B)** Representative micrograph of a longitudinal section of the central segment corresponding to the second right holobranch. (Hematoxilin–Eosin; Bar = 100 & 20 µm). **(C)** Representative micrograph of a transversal section in the middle part of the second right holobranch. (Hematoxilin–Eosin; Bar = 20 µm). (1) Primary lamella, (2) Secondary lamella, (3) Chloride cell, (4) Goblet cell, (5) Erythrocytes, (6) Pilar cell, (7) Epithelial cell, (8) Chondrocytes, (9) Extracellular cartilaginous matrix.

**Figure 3:**
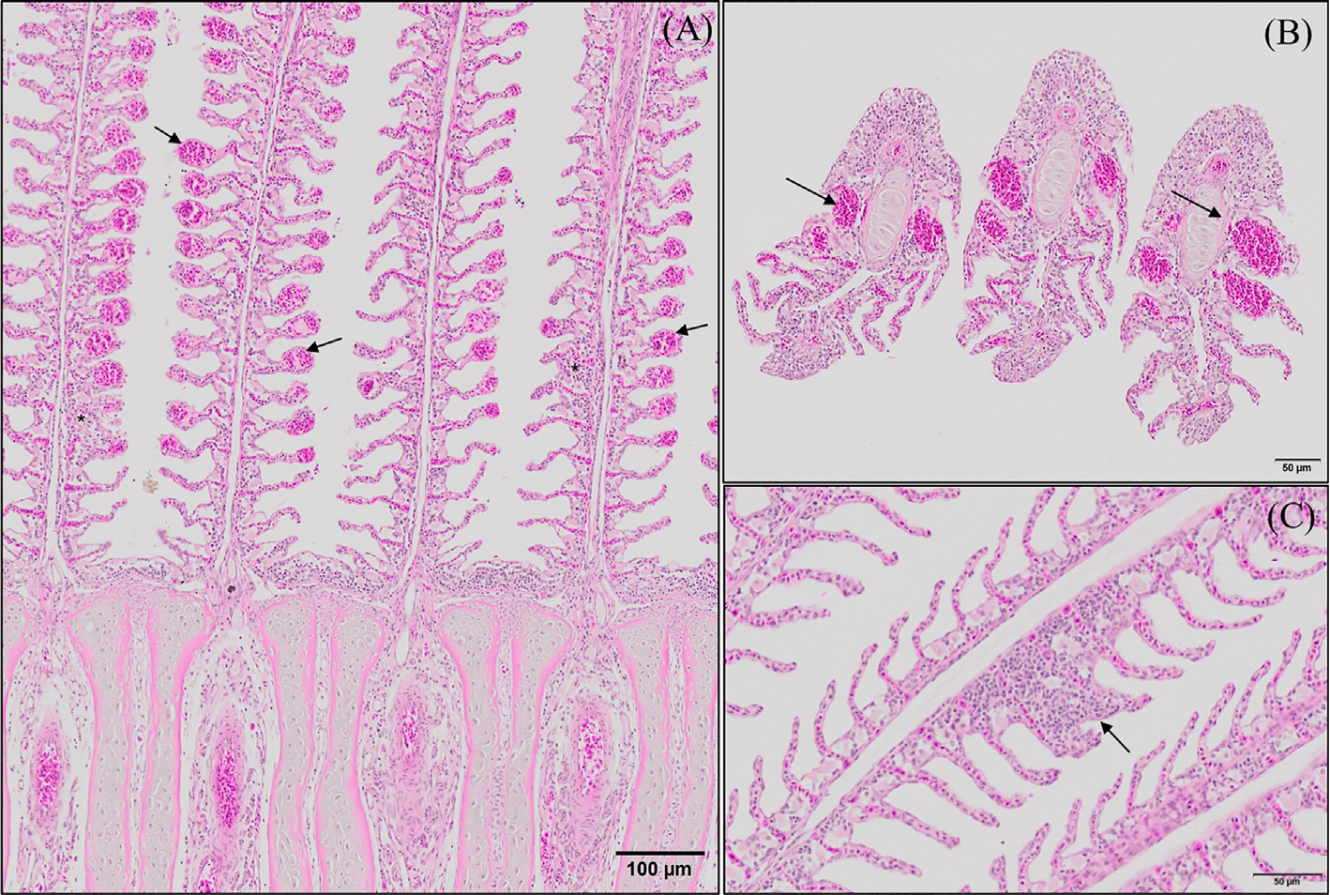
Representative micrographs of the main morphological alterations found at the end of the experimental period on European sea bass (*Dicentrarchus labrax*) gills. **(A, B)** Longitudinal and transversal sections of the primary lamellae where severe telangiectasis and/or lamellar thrombosis are indicated with arrows (Hematoxilin–Eosin; Bar = 100 & 50 µm). Focal lamellar fusion is indicated with symbols (*). **(C)** Detail of primary lamella focal lamellar fusion. Observe the filling of interlamellar space with proliferating pavement cells. (Hematoxilin–Eosin; Bar = 50 µm). Fish fed PHYTO diet presented reduced incidence (p <0.05) of lamellar fusion compared to fish fed the rest of the dietary treatments.

**Figure 4:**
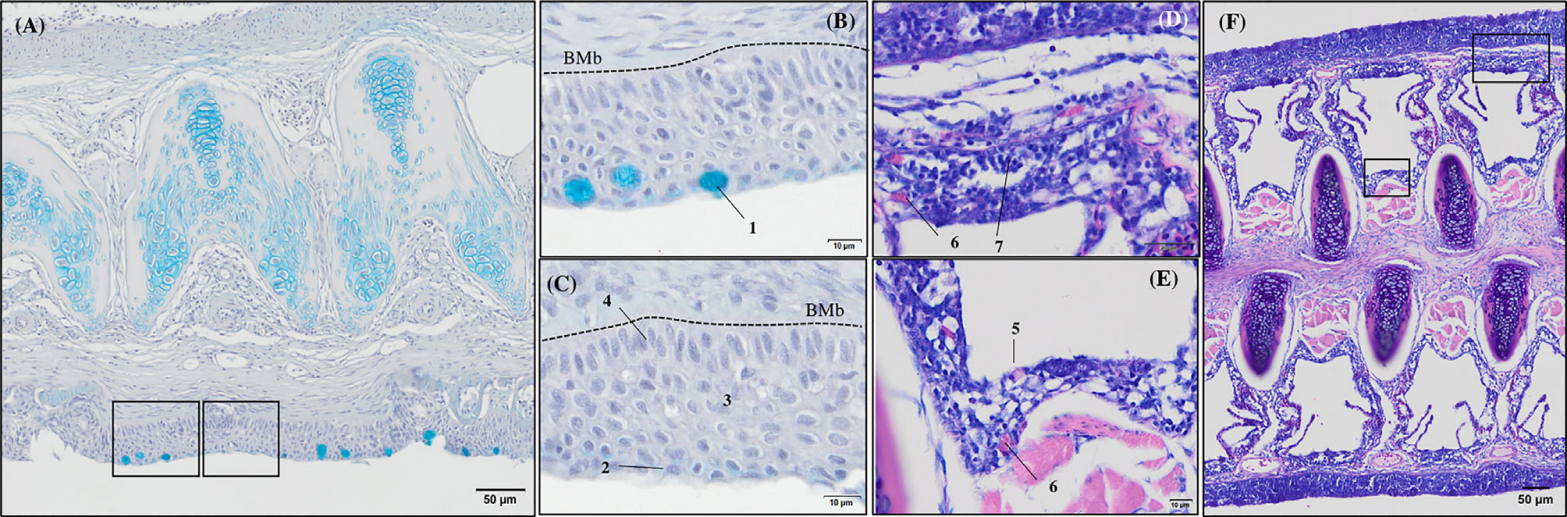
General morphology of European sea bass (*Dicentrarchus labrax*) gill base section and gill associated lymphoid tissue leucocytes populations’ distribution. **(A-C)** Representative micrograph of a transversal section of the gill base the second/third right holobranch (Alcian-Blue PAS, pH = 2.5; Bar = 50 & 10 µm). Observe the variations on the epithelial cells’ nuclei orientation. **(D-F)** Representative transversal micrograph of the interbranchial lymphoid tissue (ILT) located at the gill base the second/third right holobranch (MGG; Bar = 50 & 10 µm). (1) Goblet cell, (2) surface epithelial cells, (3) mid epithelial cells, (4) basal epithelial cells, (5) rodlet cells, (6) granulocytes, (7) lymphocytes. BMb, basement membrane.

Histopathological evaluation of H&E-/MGG-stained sagittal and transverse gill sections of segments corresponding to the second and third right holobranch revealed a well-organized secondary and primary lamellar pattern and an intact epithelial barrier in fish fed the different experimental diets (**Figure 2**). However, some morphological changes were found at the end of the experimental period, such as telangiectasis, lamellar thrombosis (**Figures 3A**, **B**), and focal lamellar fusion, characterized by filling of the interlamellar space with proliferating PVCs (**Figure 3C**). The incidence of lamellar fusion was significantly (p <0.05) lower in fish fed the PHYTO supplemented diet than that in fish fed the control diet. However, the MRC size and density and the inflammation status of the gills were not significantly (p >0.05) affected by GMOS and PHYTO, as the gills exhibited similar lymphocyte and granulocyte infiltration levels (**Table 3** and **Figures 4C–E**).

**Table 3:** Semi quantitative evaluation of the main histopathological alterations found in the gills of juvenile European sea bass (*Dicentrachus labrax*) at the end of the feeding trial (63 days).

	Dietary treatments		
	C	GMOS	PHYTO
Lamellar fusion	1-2^a^	1-2^ab^	0-1^b^
Lamellar telangiectasis/aneurysms	0-1	0-1	0
MRCs cell hyperplasia	1-2	1-2	1
General gill inflammation	1-2	1-2	1-2
ILT infiltrated leucocytes	1-2	1-2	1-2
ILT infiltrated granulocytes	1-2	1-2	1-2

1 (weak), 2 (moderate) and 3 (strong) for each parameter evaluated. Diets: C (control diet), GMOS (5000 ppm galactomannan oligosaccharides), PHYTO (200ppm phytogenic). Gill main histopathological alteration detailed in **Figure 2**. Different letters within a row denote significant differences among dietary treatments (p ≤ 0.05; Kruskal-Wallis for independent samples; U Mann-Whitney tests).

Transverse sections of the epithelial basal area of the gill arch showed clear variations in the disposition of epithelial cell nuclei in relation to the basement membrane (**Figures 4A–C**); the basal epithelial cells presented elongated cells with a central and perpendicular nuclei disposition, whereas mid epithelial cells presented a round shape with the nucleus situated parallel to the basement membrane. The surface epithelial cells presented a flat morphology with nuclei parallel to the basement membrane. Goblet cells were dispersed among the surface epithelial cells (**Figures 4B, C**). GMOS and PHYTO did not significantly affect the morphology and number of interbranchial lymphoid tissue (ITL), lymphocytes, granulocytes, and rodlet cells (**Figures 4D–F** and **Table 3**).

#### Gill Mucus Production

**Figure 5:**
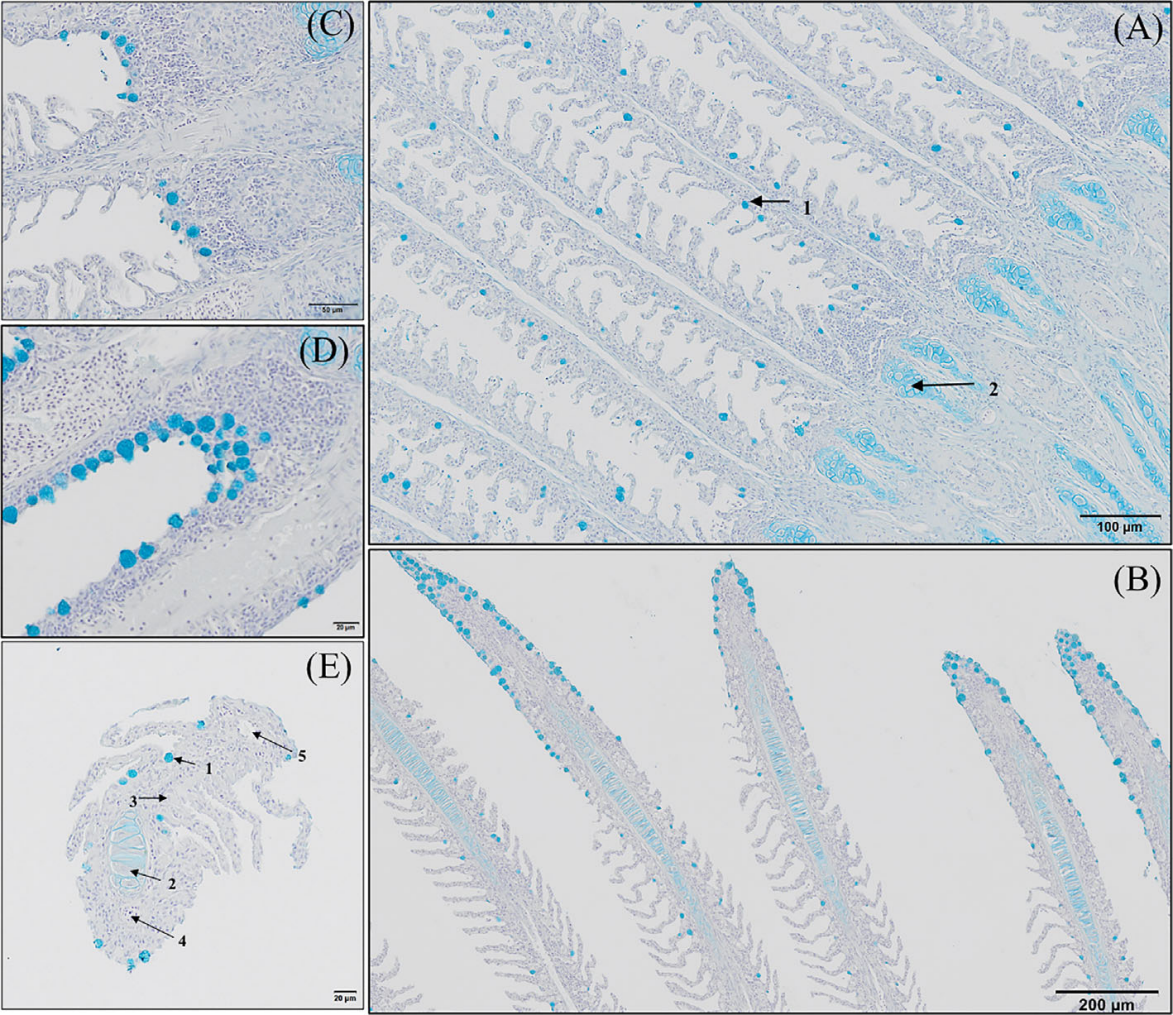
Goblet cells distribution along the gill of European sea bass (*Dicentrarchus labrax*). **(A-D)** Longitudinal section corresponding to the central segment of the second right holobranch goblet cells distribution along the primary lamella (Alcian Blue pH = 2.5). Observe how primary lamella goblet cells are mainly located between secondary lamellae (**A**; Bar = 100 µm) and in higher density surrounding the top region of the primary lamella (**B**; Bar = 300 µm). Observe the increased density of goblet cells between basal regions of the primary lamellae in contact with the interbranchial gill associated lymphoid tissue (**C**, **D**; Bar = 50 & 20 µm). **(E)** Transversal section corresponding to the central segment of the second right holobranch goblet cells

GMOS and PHYTO did not significantly affect goblet cell count and distribution. An examination of the longitudinal holobranch sections showed that goblet cells along the primary lamella were located at high density in the interlamellar spaces (between secondary lamellae) (**Figure 5A**), surrounding and protecting the top region of the primary lamella (**Figure 5B**), as well as protecting the lymphoid tissue located at the base of the primary lamellae (**Figures 5C, D**). Although the morphometric characteristics of the goblet cell was not significantly (p >0.05) affected by the supplements, the goblet cell area in the gills of fish fed the GMOS and PHYTO diets reduced by 11 and 5%, respectively, compared to the goblet cell area of the gills of fish fed the control diet (**Table 4**).

**Table 4:** Gill goblet cells morphometry of juvenile European sea bass (*Dicentrarchus labrax*) at the end of the feeding trial (63 days).

	Dietary treatments		
	C	GMOS	PHYTO
Area (µm²)	59.68 ± 4.91	53.10 ± 2.30	56.34 ± 2.94
Perimeter (µm)	38.90 ± 5.89	31.71 ± 0.72	34.47 ± 0.59
Mean radius (µm)	4.12 ± 0.20	3.93 ± 0.06	4.02 ± 0.13
Minimum diameter (µm)	6.29 ± 0.45	6.12 ± 0.09	6.11 ± 0.33

Diets: C (control diet), GMOS (5000 ppm galactomannan oligosaccharides), PHYTO (200ppm phytogenic). Morphometric studies carried out on transversal sections. Different letters within a row denote significant differences among dietary treatments (p ≤ 0.05; one-way ANOVA; Tukey).

### Gill Immunohistochemistry

**Figure 6:**
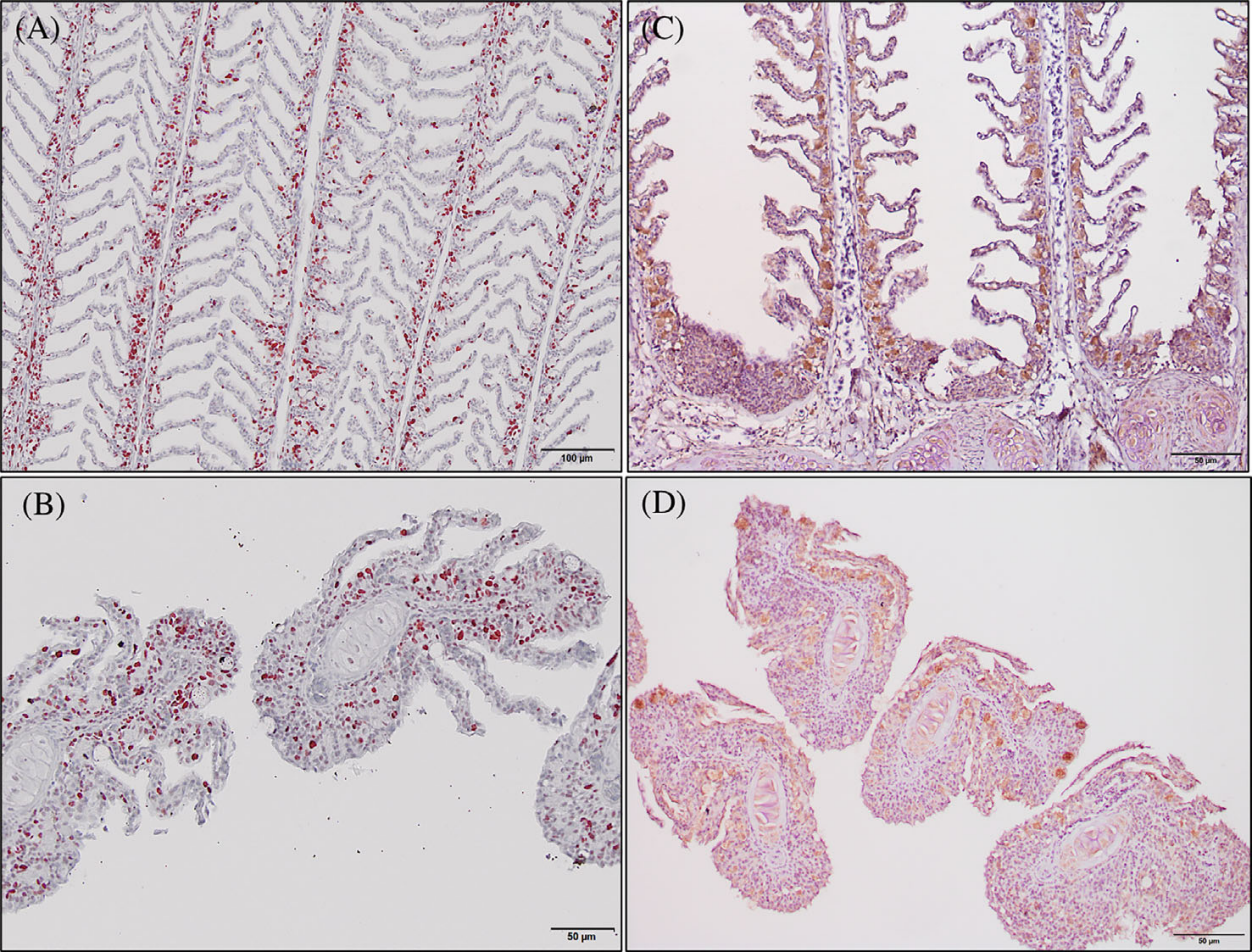
Photomicrographs of the gill immunoreactivity with the antihuman anti-PCNA and anti-iNOS-2 antibodies at the end of the feeding trial. Immunoreactivity to anti-PCNA and anti-iNOS-2 was detected in all fish gills sections studied. European sea bass gill immunoreactivity to anti-PNCA antibody (**A**, **B**; Bars = 100 and 50 µm) and to anti-INOS-2 antibody (**C**, **D**; Bar = 50 µm).

**Figure 7:**
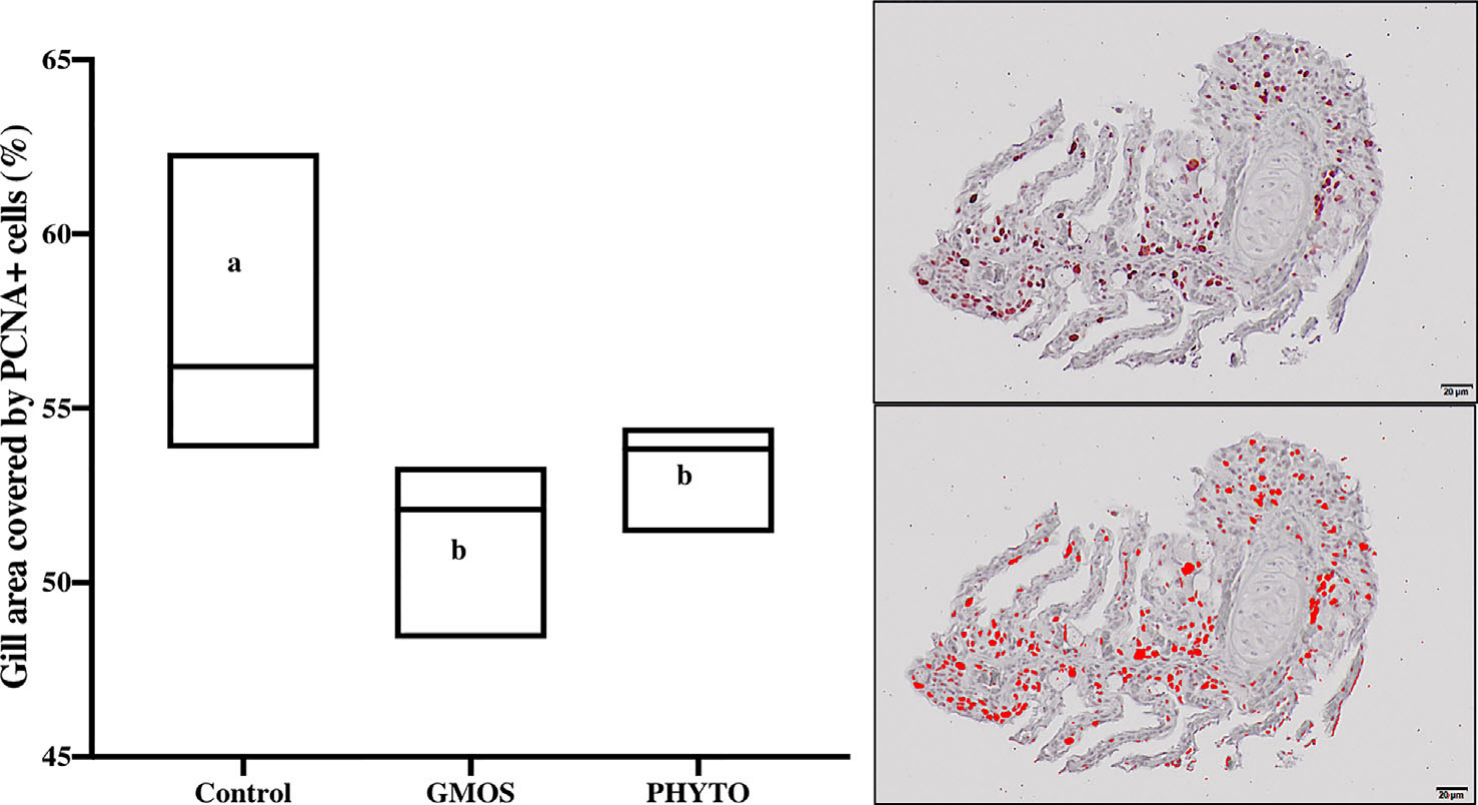
Total gill epithelium covered by PCNA+ cells on transversal sections at the end of the feeding trial (63 days). Observe as fish fed GMOS and PHYTO diets presented lower coverage of PCNA+ cells compared to fish fed control diet. Boxes in boxplot represent mean ± SD. Different letters denote significant differences among dietary treatments (p ≤ 0.05; oneway ANOVA; Tukey). Observe the automatically detected immunopositivity (marked in red) comparing the graph accompanying images.

Immunoreactivity to anti-PCNA and anti-iNOS-2 was detected in all fish gill sections studied (**Figure 6**). The presence of PCNA+ cells in the gill epithelium of fish fed the GMOS and PHYTO diets was lower than that in the gill epithelium of fish fed the control diet (**Figure 7**). The presence of anti-iNOS-2 in the gills was not significantly affected by the supplements (**Figures 6C, D**). Gill goblet cells, several leukocytes, and MCRs were immunopositive for anti-iNOS (**Figures 6C, D**).

### Gill Ultrastructure

**Figure 8:**
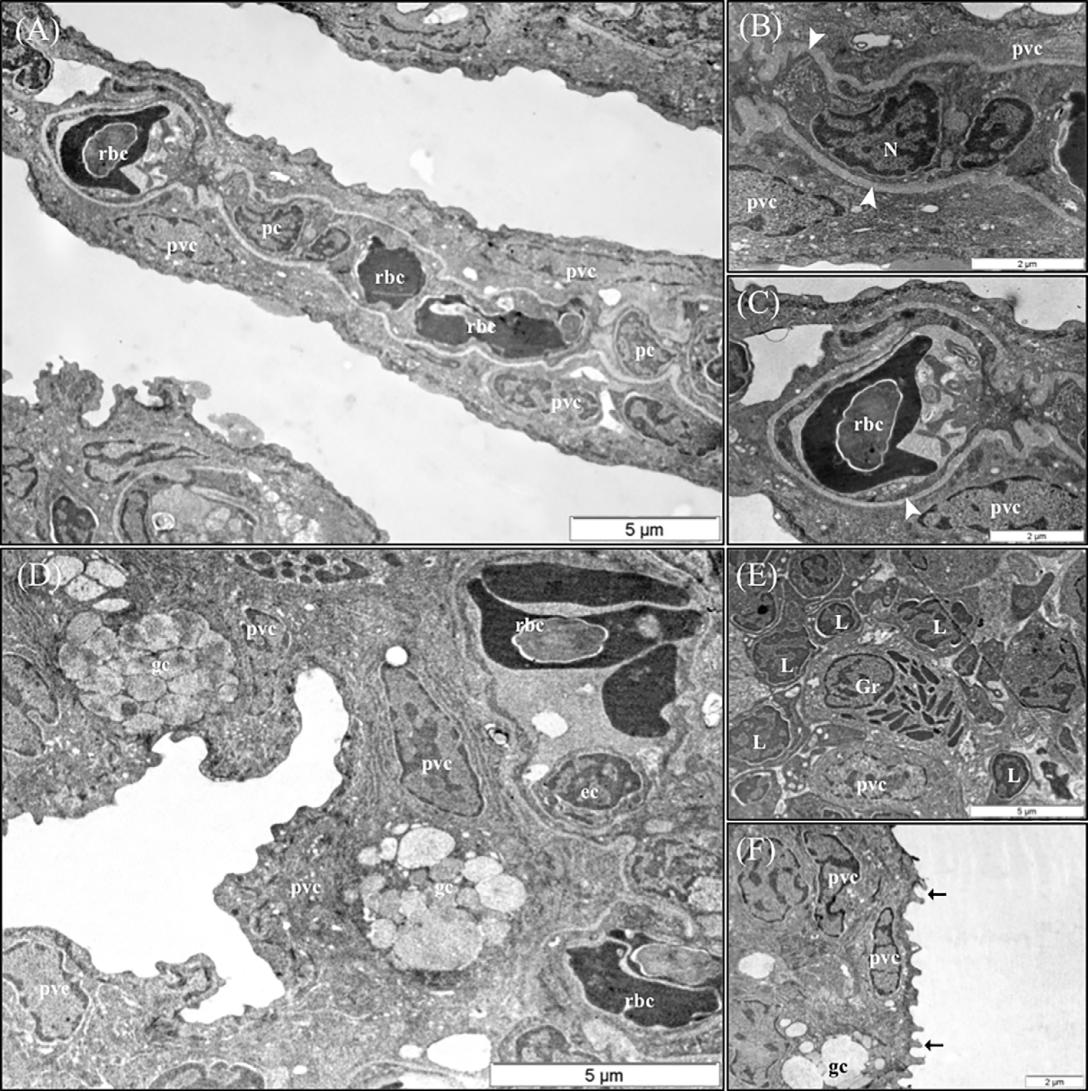
Representative TEM micrographs of the gill of European sea bass (*Dicentrarchus labrax*) fed the different dietary treatments. No significant alterations on the structural pattern of healthy gills were observed among fish fed the different dietary treatments. **(A)** Cross-section through the secondary lamellae. Observe the polymorphic nucleus of pilar cells, the electron dense red blood cells within the blood spaces and pavement cells surrounding the collagen bundle. Scale bar 5 μm. Detailed micrograph pilar cell **(B)** and blood space **(C)**, observe the collagen bundle embracing both structures (arrowhead). Scale bars 2 μm. **(D)** Pattern of the space between two secondary lamellae, where goblet cells (gc) are located and surrounded by pavement cells. Epithelial cell visible inside the blood space. Scale bar 5 μm. **(E)** Detailed micrograph of interbranchial lymphoid tissue leucocytes population. Scale bar 5 μm. **(F)** Detail of pavement cells microridges. Scale bar 2 μm. pc, Pilar cells; rbc,red blood cells; pvc, pavement cells; gc, goblet cells; ec, ephitelial cell; Gr, granulocyte; L, Lymphocyte.

**Figure 9:**
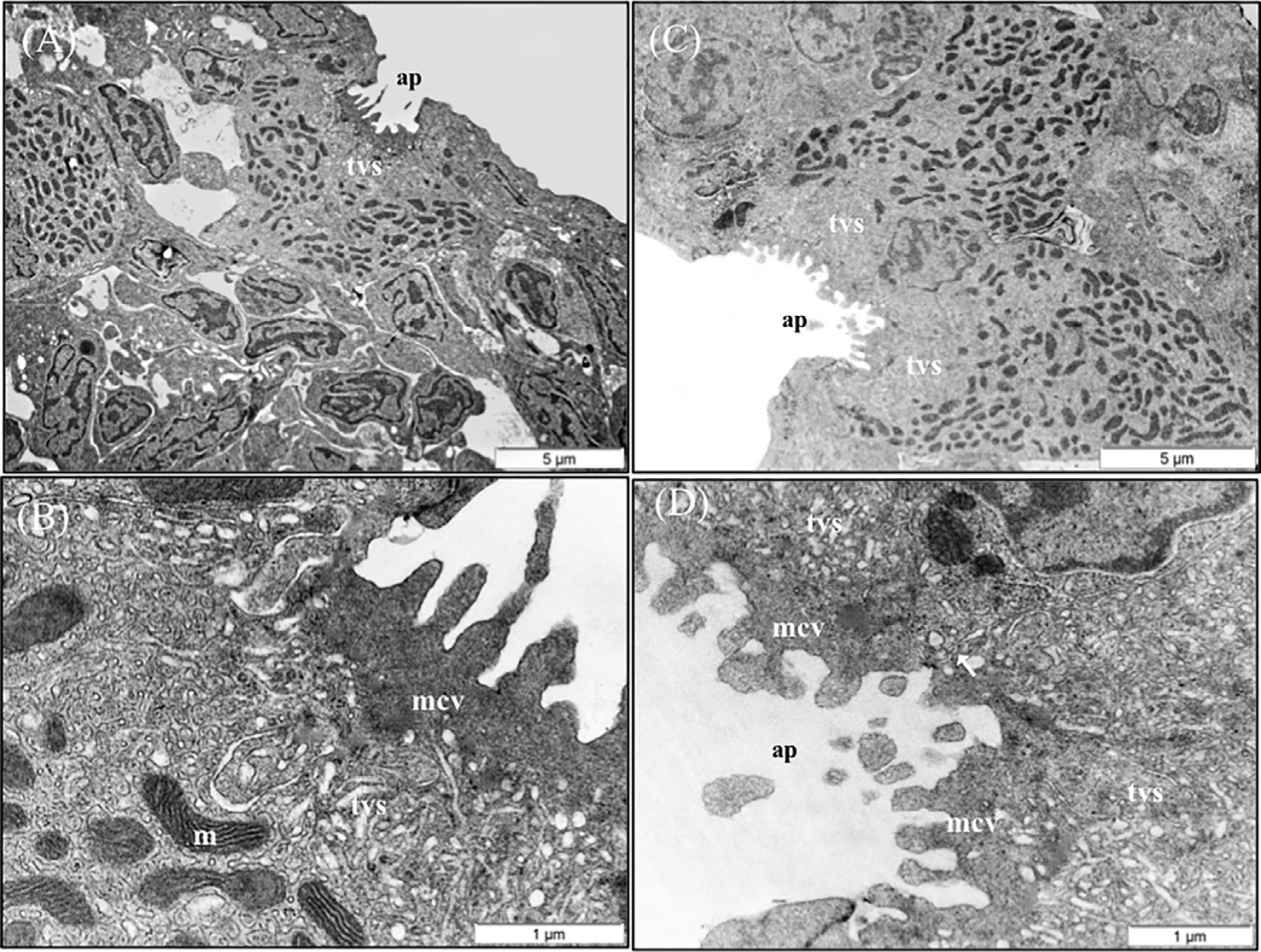
TEM micrographs of mitochondria rich cells (MRCs) present in European sea bass primary lamellae. **(A)** The crypt of MRC is usually bordered by pavement cells. Scale bar 5 μm. **(B)** Detail of the apical region of MRC, observe the electron dense microvilli, the tubulovesicular system located just below the microvilli and the mitochondria rich region in the lower region. Scale bar 1 μm. **(C)** Two MRCs sharing one apical pit. Scale bar 5 μm. **(D)** Detail of two chloride cells sharing the same apical pit. Juctional unions are indicated by arrows. tvs, Tubulovesicular system; mvc, microvilli; ap, apical pit; m, mitochondria.

The results of the qualitative TEM analysis showed that the ultrastructure of the gills was not affected by the dietary functional additives, as the gills were healthy. The gills exhibited normal appearance of secondary and primary lamellar membrane linings; normal junctional unions between cells; and normal leukocytes, pilar cells, PVCs, MRCs, and goblet cell structure and distribution. Additionally, the structure of the microridges was unaffected (**Figure 8**). Goblet cells containing secretory granules of variable electron density were in the primary lamellae, particularly in the interlamellar space between secondary lamellae (**Figure 8D**). Pilar cells were characterized by a polymorphic nucleus with condensed heterochromatin at the nuclear periphery (**Figure 8B**), and red blood cells were localized within the blood space, both being enveloped by the collagen bundle (**Figures 8A–C**) and surrounded by PVCs. Surface PVC microridges were clearly observed (**Figure 8F**). TEM analysis showed the presence of leukocytes and granulocytes in the lamellae and interbranchial lymphoid tissue (**Figure 8E**). Like the result of the histopathological examination, GMOS and PHYTO did not significantly affect the leukocyte infiltration level and distribution of the gills. **Figure 8** shows the typical structure of the MRCs, where its crypt surrounded by PVCs can be observed. MRC morphology was normal in the fish, with electron dense microvilli in the upper region and a mitochondria-rich region in the basal region. GMOS and PHYTO did not significantly affect the morphology of the tubulovesicular system of the MRCs, the intercellular union, and the appearance of the membrane lining of the evaluated cells (**Figure 9**).

## Discussion

In the present study, GMOS and PHYTO induced a significant downregulation of the expression of several genes associated with the antioxidant defense system in gills, such as *sod*, *cat*, *gpx*, and *gr*, and this may be due to an improved scavenging of excess free radicals in the gills. Previous studies have demonstrated that some prebiotics of plant origin and PFAs, such as those derived from garlic and labiate plant extracts have a potent antioxidant activity in fish (10, 40, 41, 43, 53–57). In this regard, dietary functional additives with systemic and local antioxidant capacity have been reported to act in both dose-dependent and time-dependent manner. Dietary PFAs increased fish antioxidant enzyme activity in plasma and target tissues in a dose-dependent manner, and reduced malondialdehyde (MDA) formation and tissue TBARS levels in some freshwater species, such as Nile tilapia (*Oreochromis niloticus*), rainbow trout (*Oncorhynchus mykiss*), and channel catfish (*Ictalurus punctatus*) (40, 42, 43, 58–60). Although rainbow trout juveniles fed a mixture of labiatae essential oils presented increased SOD, G6PD, and GPX activities after 30 days of feeding, the activities of CAT, GST, and GR were reduced after 60 days of dietary supplementation, while MDA concentration remained unaffected (42). A time-dependent regulation of an organism’s antioxidant capacity may be associated with self-regulation of its antioxidant system activity after reducing ROS levels. Some studies on gills have shown a discrepancy between enzymatic activity and gene expression levels (61), supporting the notion of a time-dependent self-modulation of a tissue’s antioxidant capacity, which does not always result in a positive correlation between enzymatic activity and gene expression levels.

Among the dietary supplements considered to be exogenous antioxidants or endogenous enzymatic activity enhancers of plant origin are flavonoids, plant polyphenols, allyl sulfides, and curcumin (23), some of which are present in plant extracts, garlic oil, and rich fiber extracts originating from plants and seeds. Flavonoids are phenolic substances with potent *in vitro* antioxidant properties because of their ability to reduce the formation of free radicals and scavenge free radicals. However, information available on their pharmacokinetics is limited and thus, the antioxidant activity of these compounds and their mode of action *in vivo* is poorly understood (62). Based on low plasma and tissue flavonoid concentration detected after their dietary supplementation, their significance as scavengers of free radicals was examined in higher vertebrates (63). The mode of action of flavonoids might be based on their ability to interact with several protein kinases, including mitogen-activated protein kinases (MAPKs), which is mediated *via* oxidative stress signaling cascades by several cellular reactions to vastly different stressors (63). MAPKs control the expression of endogenous antioxidant enzymes and regulate cell proliferation in several disease-associated processes, thus exerting a chemoprotective effect as modulators of oxidative stress-MAPK signaling pathways and complementing their antioxidant role scavengers of free radicals (63–66). Therefore, the observed downregulation of the expression levels of *sod, gpx, gr*, and *cat* in the gills of fish fed the supplemented diets may be partly due to the actions of GMOS and PHYTO, which regulated the endogenous antioxidant system and reduced the production of free radicals, through the MAPK signaling pathway. Furthermore, garlic derivatives, such as allyl sulfides are recognized as potent antioxidants, mainly because of their high capacity for the scavenging of free radicals (23), inhibition of leucocyte ROS production (67), control of lipid peroxidation (68, 69), and triggering of endogenous antioxidant activity (69, 70).

Protection of the gill against oxidative damage is important, as it is the site for gas exchange, osmoregulation, nitrogenous waste excretion, and pH regulation (71, 72). Furthermore, conserving the functional structure of the gill epithelium is essential for maintaining fish health, as it is continuously challenged by foreign substances and infectious agents (72). Authors of Ref. (61) examined oxidative stress in Atlantic salmon suffering from amoebic gill disease (AGD) reported reduced expression of *sod*, *cat*, and *gr* in non-affected gill areas compared with that in the affected areas (gill areas with lesion), indicating the negative effect of oxidative stress. Similarly, in the present study, we observed lower PCNA+ counts in the gills of fish fed PHYTO and GMOS supplemented diets compared with that in the gills of fish fed the control diet. Changes in the production of PCNA in the mucosal epithelium could be an early symptom of alterations in tissue homeostasis. The upregulation of the expression of gene regulating PCNA is commonly used as a marker of proliferation associated with the development of neoplastic tissue (73–76), which may be induced by exposure to external or internal stressors. Authors of Ref. (77) detected strong immunopositivity for PCNA, iNOS, HSP70, and Bax in the gills of *Hypostomus francisci* exposed to prolonged anthropogenic influences. Therefore, the reduction in PCNA counts in the gills of the fish fed PHYTO and GMOS supplemented diets might be associated with reduced levels of oxidative species *via* a modulation of the endogenous antioxidant capacity. Garlic extract, such as flavonoids, ameliorated oxidative stress in rat cardiomyocytes by reducing the production of NO and H_2_S (78, 79). Although GMOS and PHYTO did not significantly affect the iNOS concentration and inflammation status of the gills in the present study, in a parallel study (79), reported that GMOS and PHYTO at the same dose and test duration with that of the present study mitigated leucocytic apoptotic processes associated with stress in European sea bass at the genetic level. Fish fed the PHYTO supplemented diet had lower level of lamellar fusion and a tendency to reduce the incidence of lamellar telangiectasis and MRC hyperplasia. Although the distribution of goblet cells in the primary lamellae was unaffected by the supplements, fish fed GMOS supplemented diet presented a smaller goblet cell area compared with that in the control group, and this agrees with the results of previous studies. Authors of Ref. (52) reported reduced intestinal goblet cell hyperplasia in European sea bass fed low FM/FO diets supplemented with GMOS and PHYTO. The change in goblet cell area may be because of an induced dysbiosis, which was produced by low dietary FM/FO diets (80). However, direct changes in the composition and consistency of gill mucus after functional diet supplementation, which may protect individual secondary lamellae from adhesion, should not be discarded because any potential change in the composition of gill mucus may affect the dynamics of pathogen invasion and persistence in the host (81).

In this regard, cell junctions play an important role in protecting the host’s mucosal health (81, 82); however, as in other cultured fish species, the regulation of the functions of gill junctional complexes in European sea bass, particularly the roles of functional additives, is not well understood. Among the regulators of tight junctions are *zo-1* and *ocln*. In the present study, the expression of *ocln* was downregulated by GMOS, whereas *zo-1* showed an increasing trend; however, the opposite tendency was observed in fish fed PHYTO diet. The expression of certain genes related to the tight junction complex is involved in the response to environmental stressors, such as hypoxic stress (83–85) or pathogen exposure (82). In the present study, the gills of fish fed the functional diets had higher *zo-1* and *hsp70* expression levels and lower expression of *ocln.* In a similar study, we observed a downregulation of the expression of hypoxia inducible factor *(hif-1)*, lower cortisol levels, and a reduction in apoptosis in head kidney leucocytes after 2 h of stress by confinement in fish fed GMOS and PHYTO diets (79). An increase in the deposition of *ocln* and specific *claudins* was observed in the tight junctions of single-seeded insert cultures of PVCs from puffer fish (*Tetraodon nigroviridis*) in response to cortisol (86), thus, conditioning the permeability of the paracellular pathway. Variations in the expression of genes related to tight junction complexes might affect both the cellular reorganization of the cytoskeleton and bacterial binding (85).

GMOS and PHYTO dietary supplementation did not exert a clear effect on the morphology and density of the ITL-infiltrated leucocytes on a qualitative basis, the anti-inflammatory role of PFAs and GMOS and their effects on the ITL cytokine microenvironment and possible mechanisms involved on cytoskeleton rearrangement should be considered in future studies.

In conclusion, our findings indicated that dietary GMOS (5,000 ppm) and PHYTO (200 ppm) in 10%FM/6%FO-based diets protected the gill epithelia of European sea bass from oxidative stress by modulating the expression patterns of oxidative enzyme-related genes and lowering PCNA+ cell count. Moreover, PHYTO supplementation reduced lamellar fusion, whereas GMOS supplementation reduced goblet cell count by 11%, but did not affect the distribution goblet cells along the primary lamellae. These protective effects should be considered in future prevention strategies against gill mucosal diseases in fish species, especially those sensitive to gill-associated pathogens. Further studies should be conducted to establish the modes of action that are affected by each functional product under different scenarios of oxidative stress.

## Data Availability Statement

The datasets presented in this study can be found in online repositories. The names of the repository/repositories and accession number(s) can be found in the article/**Supplementary Material**.

## Ethics Statement

The Bioethical Committee of the University of Las Palmas de Gran Canaria approved all the protocols used in the present study (approval no. 007/2012 CEBA ULPGC).

## Author Contributions

ST, MI, FA, and DM contributed to the conception and design of the study. ST performed formal analysis. ST, AS, and GT performed the statistical analyses. ST wrote the first draft of this manuscript. ST, MI, FA, and DM acquired funding. Resources were provided by ST, DM, VV-V, GT, and AM. All authors contributed to the article and approved the submitted version.

## Funding

This work was funded by the Spanish Ministry of Economy, Industry and Competitiveness (“Subprograma Estatal de Generación de Conocimiento, en el marco del Plan Estatal de Investigación Científica y Técnica y de Innovación 2013-2016”) by the PROINMUNOIL PLUS (AGL2016-79725-P) project: “Functional diets for marine raw materials replacement: boosting the fish disease resistance through epithelial barriers reinforcement and immunization tools,” in which DM, ST, FA, MI and AS were involved. GT was responsible for the gene expression analyses, which were funded by the EU Horizon 2020 AquaIMPACT (Genomic and nutritional innovations for genetically superior farmed fish to improve efficiency in European aquaculture), number: 818367. Besides, the Spanish Ministry of Economy, Industry, and Competitiveness funded ST research though the “Subprograma Juan de la Cierva-Incorporación, Convocatoria 2015, IJCI2015-25748.

## Conflict of Interest

Author AM and VV-V were employed by company Delacon Biotechnik GmbH and Biomar A/S, respectively.

The remaining authors declare that the research was conducted in the absence of any commercial or financial relationships that could be construed as a potential conflict of interest.
