# Supplementary material for: Dietary Phytogenics and Galactomannan Oligosaccharides in Low Fish Meal and Fish Oil-Based Diets for European Sea Bass (Dicentrarchus labrax) Juveniles: Effects on Gill Structure and Health and Implications on Oxidative Stress Status
Source: Front Immunol. 2021 May 12;12:663106. doi: 10.3389/fimmu.2021.663106 (PMC8149968; doi:10.3389/fimmu.2021.663106)

Supplementary Figure 1. Calculation of the efficiency (E) of each primer set used in the study. The CFX Maestro™ Software (Biorad) has been used to calculate the efficiency and to select the appropriate reference gene and analyze its stability by means of the reference gene selection tool (please see CFX Maestro™ Software User Guide Version 1.1).

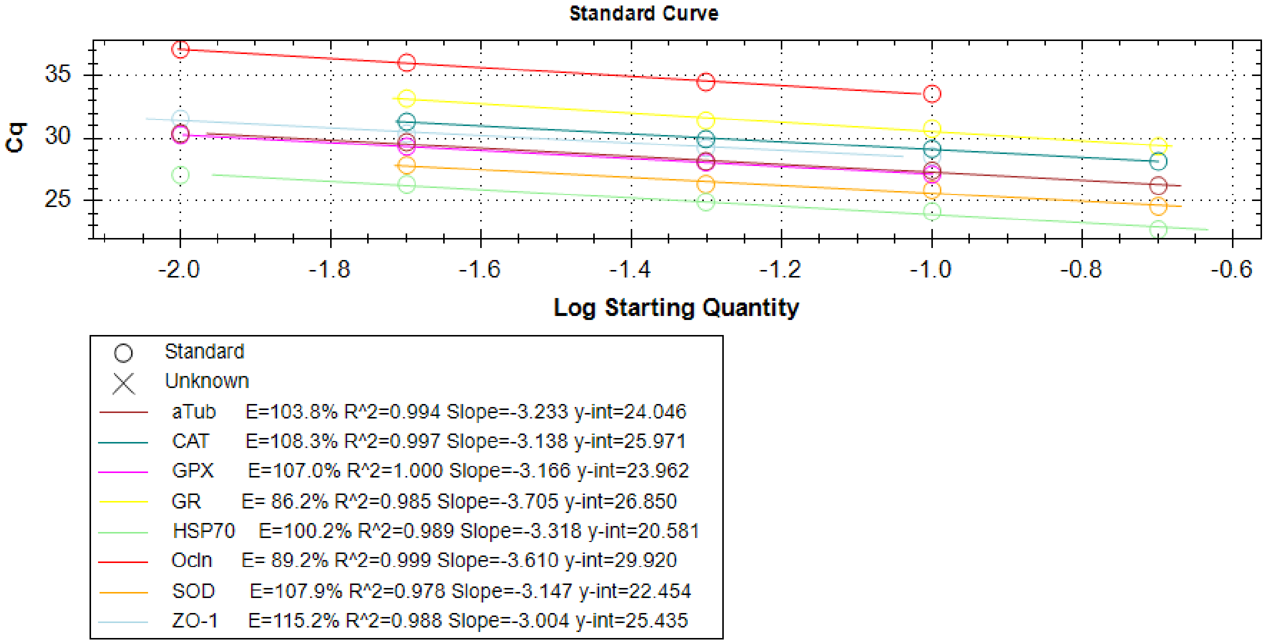

Supplement: Supplementary file 1 [file DataSheet_1.pdf]
